# Efficient liver repopulation of transplanted hepatocyte prevents cirrhosis in a rat model of hereditary tyrosinemia type I

**DOI:** 10.1038/srep31460

**Published:** 2016-08-11

**Authors:** Ludi Zhang, Yanjiao Shao, Lu Li, Feng Tian, Jin Cen, Xiaotao Chen, Dan Hu, Yan Zhou, Weifen Xie, Yunwen Zheng, Yuan Ji, Mingyao Liu, Dali Li, Lijian Hui

**Affiliations:** 1 State Key Laboratory of Cell Biology, Shanghai Institute of Biochemistry and Cell Biology, Shanghai Institutes for Biological Sciences, Chinese Academic of Sciences, Shanghai, China; 2 Shanghai Key Laboratory of Regulatory Biology, Institute of Biomedical Sciences and School of Life Sciences, East China Normal University, Shanghai, China; 3 Department of Pathology, Zhongshan Hospital, Fudan University, Shanghai, China; 4 State Key Laboratory of Bioreactor Engineering, School of Bioengineering, East China University of Science and Technology, Shanghai, China; 5 Department of Gastroenterology, Changzheng Hospital, Second Military Medical University, Shanghai, China; 6 Department of Advanced Gastroenterological Surgical Science and Technology, Faculty of Medicine, University of Tsukuba, Tsukuba, Japan

## Abstract

Hereditary tyrosinemia type I (HT1) is caused by a deficiency in the enzyme fumarylacetoacetate hydrolase (Fah). Fah-deficient mice and pigs are phenotypically analogous to human HT1, but do not recapitulate all the chronic features of the human disorder, especially liver fibrosis and cirrhosis. Rats as an important model organism for biomedical research have many advantages over other animal models. Genome engineering in rats is limited till the availability of new gene editing technologies. Using the recently developed CRISPR/Cas9 technique, we generated *Fah*^−/−^ rats. The *Fah*^−/−^ rats faithfully represented major phenotypic and biochemical manifestations of human HT1, including hypertyrosinemia, liver failure, and renal tubular damage. More importantly, the *Fah*^−/−^ rats developed remarkable liver fibrosis and cirrhosis, which have not been observed in *Fah* mutant mice or pigs. Transplantation of wild-type hepatocytes rescued the *Fah*^−/−^ rats from impending death. Moreover, the highly efficient repopulation of hepatocytes in *Fah*^−/−^ livers prevented the progression of liver fibrosis to cirrhosis and in turn restored liver architecture. These results indicate that *Fah*^−/−^ rats may be used as an animal model of HT1 with liver cirrhosis. Furthermore, *Fah*^−/−^ rats may be used as a tool in studying hepatocyte transplantation and a bioreactor for the expansion of hepatocytes.

Hereditary tyrosinemia type I (HT1) is an autosomal recessive inborn error of metabolism that is caused by defects in the enzyme, fumarylacetoacetate hydrolase (Fah). Fah is an enzyme required in the last step of the tyrosine catabolic pathway, which hydrolyzes fumarylacetoacetate into fumarate and acetoacetate^1^. A deficiency in Fah leads to the accumulation of toxic metabolites, including fumarylacetoacetate and maleylacetoacetate^2^. HT1 affects the liver and kidney, which are organs where Fah is mainly expressed^3^,^4^. The acute presentation of HT1 is with fatal liver failure in infancy, and its chronic presentation is with cirrhosis and hepatocellular carcinoma (HCC) early in childhood^5^. Renal tubular damage in HT1 patients generally leads to Fanconi syndrome, developmental hypophosphatemic rickets, and growth failure^6^. When left untreated, most HT1 patients die of acute and severe hepatorenal failure during early infancy^5^. 2-(2-Nitro-4-trifluoromethylbenzoyl)-1,3-cyclohexanedione (NTBC) as an inhibitor of 4-hydroxyphenylpyruvate dioxygenase acts by blocking the accumulation of toxic metabolites such as fumarylacetoacetate and maleylacetoacetate (Supplementary Fig. 1)^7^. NTBC treatment is effective in ameliorating liver and kidney damage in HT1 patents. Combined with a low tyrosine diet, it has achieved >90% survival rate^8^,^9^. However, some HT1 patients do not respond to NTBC, thereby rendering liver transplantation as the only option to date^10^.

The *Fah* gene is conserved in mammals. High homology has been observed among human, pig, rat, and mouse cDNAs (80% homology) and proteins (94% homology)^4^. Fah-deficient mice and pigs have previously been reported as disease models for HT1^11^,^12^,^13^,^14^,^15^. Fah-deficient mice and pigs are maintained using NTBC treatment, and both models develop progressive liver failure and renal tubular dysfunction after NTBC withdrawal^12^,^16^. Notably, *Fah*^−/−^ mice can be rescued by wild-type (WT) hepatocyte transplantation, with almost complete liver repopulation by transplanted cells^16^,^17^. This approach makes them also a valuable model for evaluating the *in vivo* functions of lately identified liver progenitor cells and hepatocyte-like cells, a model for generating to generate humanized liver and a bio-factory for the *in vivo* expansion of human hepatocytes^18^,^19^,^20^,^21^,^22^,^23^. Interestingly, *Fah*^−/−^ mice can also be used to assess the therapeutic efficacy of transplanted cells from extrahepatic sources such as bone marrow or adult pancreas^24^,^25^. Nevertheless, both mouse and pig models fail to recapitulate key chronic manifestations of HT1 patients, namely, liver fibrosis and cirrhosis^13^.

Rats, as a medium-sized laboratory model, are at least 10 times larger than mice, and thus are capable of providing greater blood volumes and more bile acid, cells, and tissues for analyses^26^. More importantly, rats are more similar to humans than mice in terms of various physiological and pathological aspects. For example, rats recapitulate a more accurate phenotype for inflammatory disorders and neurodegenerative diseases, such as Parkinson’s disease and Huntington’s disease^27^. Interestingly, rats generally show more liver fibrosis than mice upon toxin-induced liver injury^28^. Furthermore, rats are routinely employed in testing drug therapeutic efficacy and toxicity before human clinical trials^29^, and abundant physiological and pharmacological data from rats has been accumulated. However, the absence of genetic modification technologies in rats has largely impeded its application in biomedical studies in the last three decades. While our manuscript was under preparation, a *Fah*^−/−^*IL2rg*^−/−^ rat model was reported for the evaluation of the *in vivo* functions of rat liver stem cells^30^. However, detailed characterization of human HT1 manifestations in *Fah*^−/−^ rats remains unclear. We are one of the first groups to adapt the CRISPR/Cas9 system to generate genetically modified rats^31^,^32^,^33^. In the present study, we generated *Fah*^−/−^ rats using the CRISPR/Cas9 system. *Fah*^−/−^ rats were maintained with drinking water supplemented with NTBC. After NTBC withdrawal, the *Fah*^−/−^ rats developed majority of human HT1 manifestations, including liver fibrosis and cirrhosis. Importantly, WT hepatocyte transplantation improved the survival of *Fah*^−/−^ rats via efficient liver repopulation as well as prevented the development of liver cirrhosis.

## Results

### Generation of heritable *Fah* mutant rats using the CRISPR/Cas9 system

**Figure 1:**
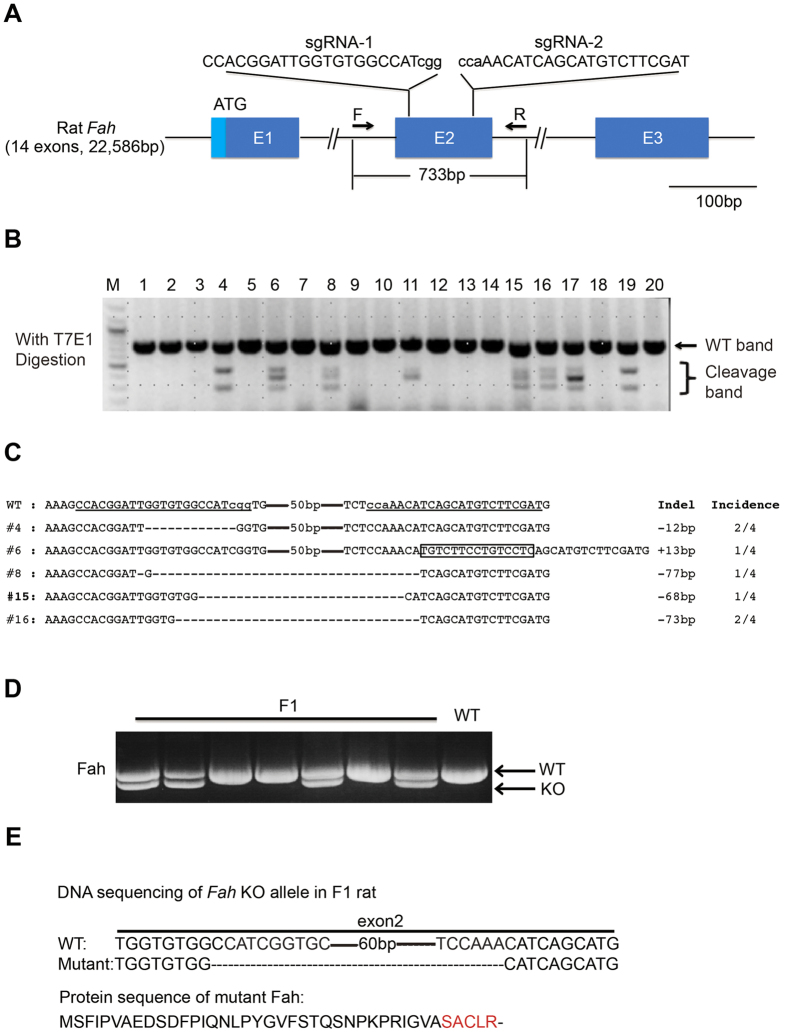
Generation of heritable Fah mutant rats by using the CRISPR/Cas9 system. (**A**) Schematic representation of a part of the rat *Fah* gene. The magnified views illustrate the binding sites of the sgRNAs. F and R represent the forward and reverse primers used in genotyping. (**B)** Detection of mutations in F_0_ rats by T7E1 digestion using PCR products amplified from tail genomic DNA. Cleavage bands indicate the presence of mutations in the *Fah* gene in F_0_ rats. (**C**) DNA sequence of the *Fah* gene in founders. Four TA clones of the PCR products amplified from each founder were analyzed by DNA sequencing. sgRNA sequences are underlined. The short dash lines and black boxes indicate deletion and insertion of nucleotides, respectively. Changes in DNA sequence are shown at right. The incidence of each genotype in the four clones is listed in the rightmost column. (**D**) The genotypes of the F_1_ rats were determined by PCR analysis. (**E**) The DNA and protein sequences of the mutant *Fah* gene in F_1_ rats are identical to those in founder #15. The amino acids in red, caused by frame-shifting mutations, differ from that of the wild-type (WT) Fah protein sequence.

To generate a *Fah*-null allele, two single guide RNAs (sgRNAs) targeting exon 2 of the rat *Fah* gene (Fig. 1A) were co-injected with Cas9 mRNA into one cell-stage Sprague-Dawley (SD) rat embryos. The manipulated embryos were then transferred into pseudopregnant females. The genomic DNA of the newborns was extracted from tail-tips for PCR amplification using primers flanking the target locus. After T7 endonuclease I (T7EI) digestion, 8 out of 20 pups were identified as founders, bearing mutations in the *Fah* gene (Fig. 1B). DNA sequencing of the 5 founders confirmed that these were heterozygotes with insertion/deletion (indel) mutations in one allele. 4 out of the 5 indel mutations caused frame-shifting mutations and premature termination (Fig. 1C). Among these, the mutation in founder #15 was predicted to generate the shortest Fah truncation protein. We bred founder #15 with WT rats. Genome PCR (Fig. 1D) and DNA sequencing (Fig. 1E) confirmed that the same *Fah* mutation was present in the F_1_ rats, thereby suggesting that this mutated *Fah* allele is heritable.

One important concern relating to genome editing with CRISPR/Cas9 is the occurrence of potential off-target effects. We predicted the potential off-target sites of *Fah* sgRNAs in the rat genome based on a published software tool (Supplementary Table 1)^34^. The three top-ranking off-target sites for each sgRNA were cloned and sequenced. No mutations were observed at these potential off-target sites in founder #15. These results were in line with the findings of previous studies that few off-target effects were induced via embryonic injection of the CRISPR/Cas9 system^32^,^35^. To further exclude unknown off-target sites, founder #15 was backcrossed to WT rats for two generations, and *Fah*^+/−^ rats were then bred to homozygotes.

### NTBC treatment prevents neonatal death of *Fah* ^−/−^ rats

**Figure 2:**
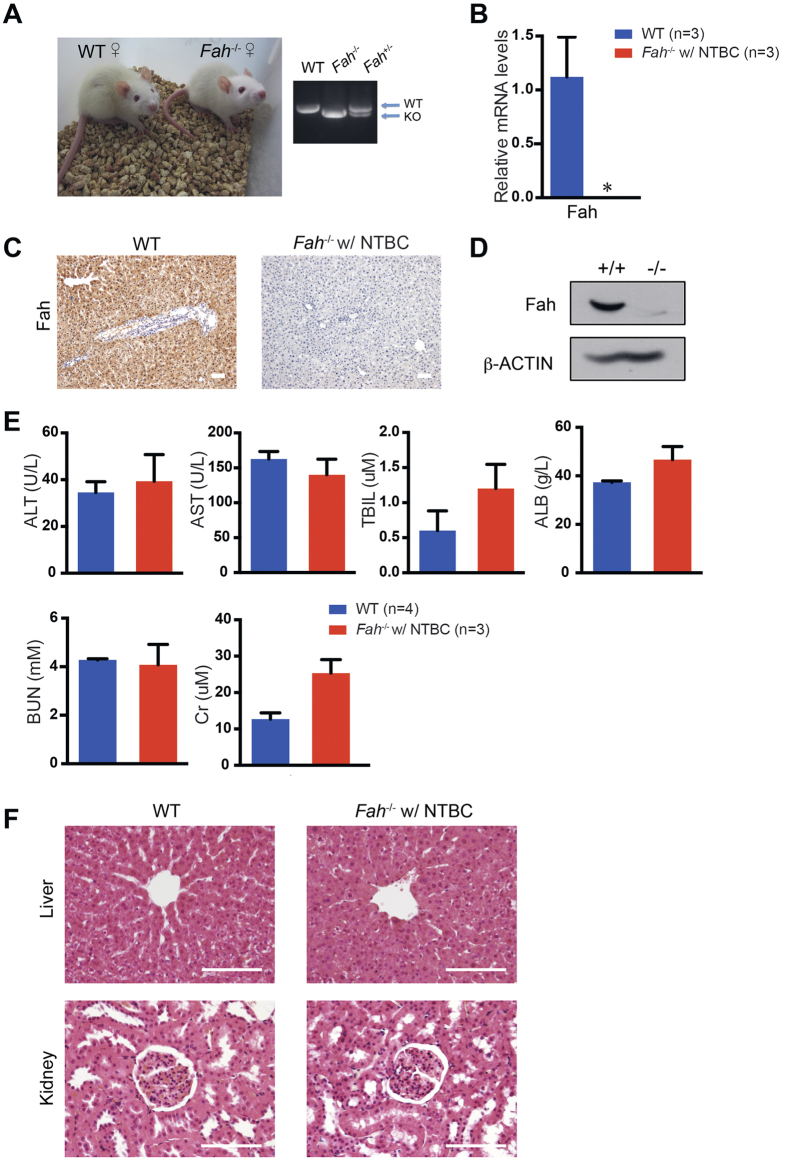
NTBC treatment prevents neonatal death in *Fah*^−/−^ rats. *Fah*^+/−^ rats were bred to obtain homozygotes. NTBC was administrated during gestation and after birth. (**A**) Four-week-old female wild-type (WT) and *Fah*^−/−^ rats treated with NTBC (*Fah*^−/−^ w/ NTBC). Genotyping was performed by PCR. (**B–**) Relative mRNA expression levels (**B**), immunohistochemistry (IHC) staining (**C**) and Western blotting (D) of *Fah* in the livers of four-week-old WT (n = 3) and *Fah*^−/−^ (n = 3) rats treated with NTBC. *P < 0.01, *t*-test. (**E**) Serum ALT, AST, TBIL, ALB, BUN, and Cr levels of 8-week-old WT (n = 4) and *Fah*^−/−^ (n = 3) rats treated with NTBC. (**F**) The hematoxylin and eosin (H&E) staining of livers and kidneys in four-week-old WT and *Fah*^−/−^ rats treated with NTBC. Scale bars, 100 μm.

The *Fah*^+/−^ rats were healthy and apparently showed no defects. Serum biochemical analyses detected no liver and kidney dysfunction in the *Fah*^+/−^ rats (Supplementary Fig. 2A). The *Fah*^−/−^ rats were born from heterozygous *Fah*^+/−^ rats following the predicted Mendelian ratio. However, none of the *Fah*^−/−^ newborns survived longer than three days after birth in the absence of NTBC. Upon NTBC addition to the drinking water, the *Fah*^−/−^ rats underwent normal growth and were indistinguishable from their WT littermates (Fig. 2A). Notably, the complete loss of the Fah protein in *Fah*^−/−^ rats was confirmed by quantitative PCR(q-PCR) analysis, immunohistochemical staining and Western blotting of Fah (Fig. 2B–D and Supplementary Fig. 2B). The *Fah*^−/−^ rats on NTBC displayed normal body weight, whereas their liver-body weight ratio was slightly increased (Supplementary Fig. 2C,D). To determine whether NTBC prevented hepatic and renal damage in *Fah*^−/−^ rats, blood biochemical parameters relative to liver and kidney functions were measured in 8-week-old rats. The serum levels of albumin (ALB), alanine transaminase (ALT), aspartate aminotransferase (AST), total bilirubin (TBIL), blood urea nitrogen (BUN), and creatinine (Cr) were within the normal ranges in both *Fah*^−/−^ rats on NTBC and WT control rats (Fig. 2E). These biochemical measurements were indicative of normal liver and kidney functions in *Fah*^−/−^ rats on NTBC. Histological analysis also revealed that the *Fah*^−/−^ rats maintained normal liver and kidney tissue architectures with NTBC treatment (Fig. 2F). We did not observe apparent sickness in *Fah*^−/−^ rats after more than one year of NTBC exposure (Supplementary Fig. 3). Taken together, these findings suggest that NTBC protects *Fah*^−/−^ rats from neonatal death caused by a deficiency in Fah.

### *Fah* ^−/−^ rats develop liver failure and cirrhosis after NTBC withdrawal

**Figure 3:**
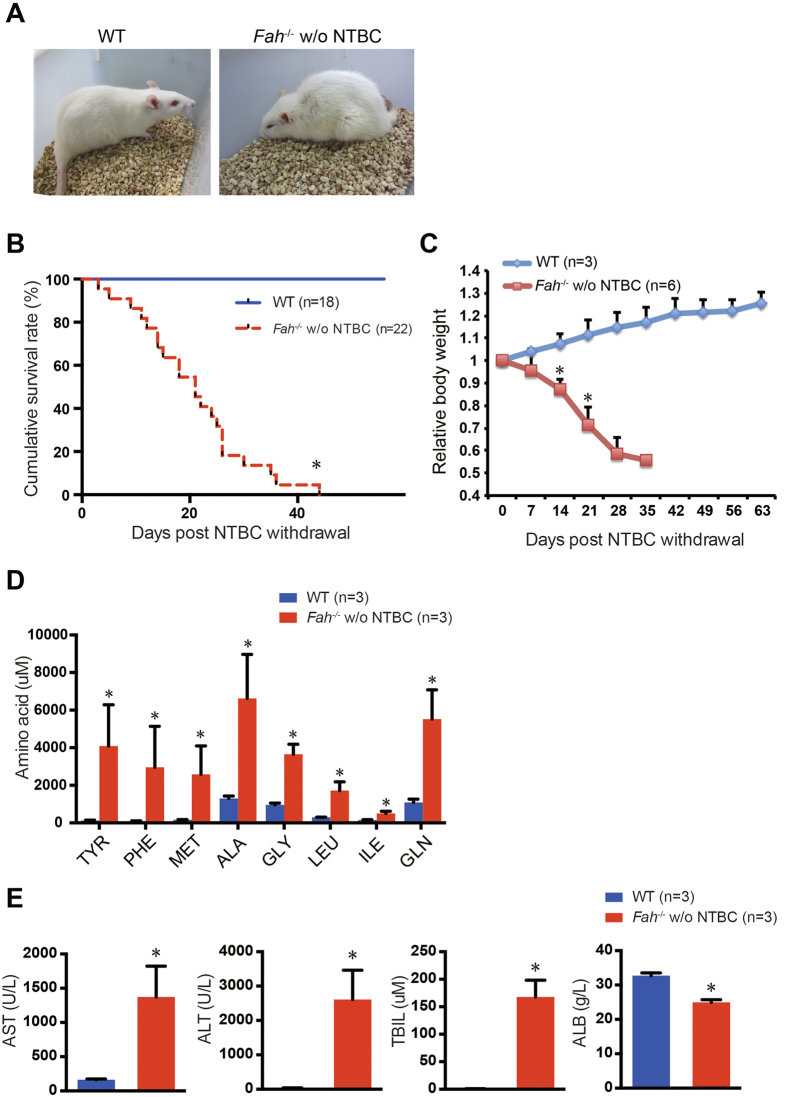
*Fah*^−/−^ rats rapidly undergo clinical deterioration due to liver dysfunction after NTBC withdrawal. *Fah*^−/−^ rats were exposed to NTBC for two months after birth. NTBC was then removed to mimic the manifestations of human HT1. (**A**) WT and *Fah*^−/−^ rats off NTBC (*Fah*^−/−^ w/o NTBC) for 3 weeks. (**B**) Kaplan-Meier survival curve of wild-type (WT) (n = 18) and *Fah*^−/−^ (n = 22) rats after NTBC removal. *P < 0.001, log-rank test. (**C**) The body weights of the WT (n = 3) and *Fah*^−/−^ (n = 6) rats were measured every week after NTBC removal. The data are respectively normalized to their body weights at Day 0. *P < 0.01, *t*-test. (**D**) Serum amino acid levels of WT (n = 3) and moribund *Fah*^−/−^ (n = 3) rats within 3 weeks after withdrawal of NTBC. *P < 0.05, *t*-test. (E) Serum ALB, AST, ALT, and TBIL levels in WT (n = 3) and moribund *Fah*^−/−^ (n = 3) rats within 3 weeks after withdrawal of NTBC. *P < 0.01, *t*-test.

We next analyzed the manifestations and visceral pathologies of HT1 in *Fah*^−/−^ rats after NTBC withdrawal. *Fah*^−/−^ rats were maintained with NTBC in drinking water for 8–10 weeks after birth. NTBC was then discontinued until the *Fah*^−/−^ rats were moribund and sacrificed (Fig. 3A). Most of the rats (18/22, 81%) died within 4 weeks after NTBC withdrawal, whereas the rest rats (4/22) eventually died within 7 weeks after NTBC withdrawal (Fig. 3B). Compared to WT rats, *Fah*^−/−^ rats failed to thrive and lost 40% of their body weight at 4 weeks after NTBC withdrawal (Fig. 3C). Elevated serum levels of tyrosine, phenylalanine, and methionine caused by the defective metabolism of amino acids are commonly observed in HT1 patients^36^. Accordingly, the tyrosine level of moribund *Fah*^−/−^ rats within 3 weeks after NTBC withdrawal was elevated 32-fold compared to that of WT rats, thereby indicating hypertyrosinemia (Fig. 3D). Elevated serum levels for the other amino acids were also observed in the *Fah*^−/−^ rats after NTBC withdrawal (Fig. 3D). The liver function of these moribund *Fah*^−/−^ rats was further assessed by blood biochemistry analysis. The serum levels of ALT, AST, and TBIL increased to a significantly high level, whereas that of ALB decreased by 25% (Fig. 3E), thereby suggesting severe impaired liver function in these rats. No significant sex differences in NTBC-off *Fah*^−/−^ rats were observed based on a comparison of survival curve and liver injury (Supplementary Fig. 4A,B). Accumulation of fumarylacetoacetate leads to profound DNA damage in livers of *Fah*^−/−^ mice, thereby causing the deregulated gene expression^37^. Indeed, the expression levels of DNA damage response and cell cycle arrest related genes, including CHOP, p53 and p21, were dramatically increased in NTBC-off *Fah*^−/−^ livers (Supplementary Fig. 4C).

**Figure 4:**
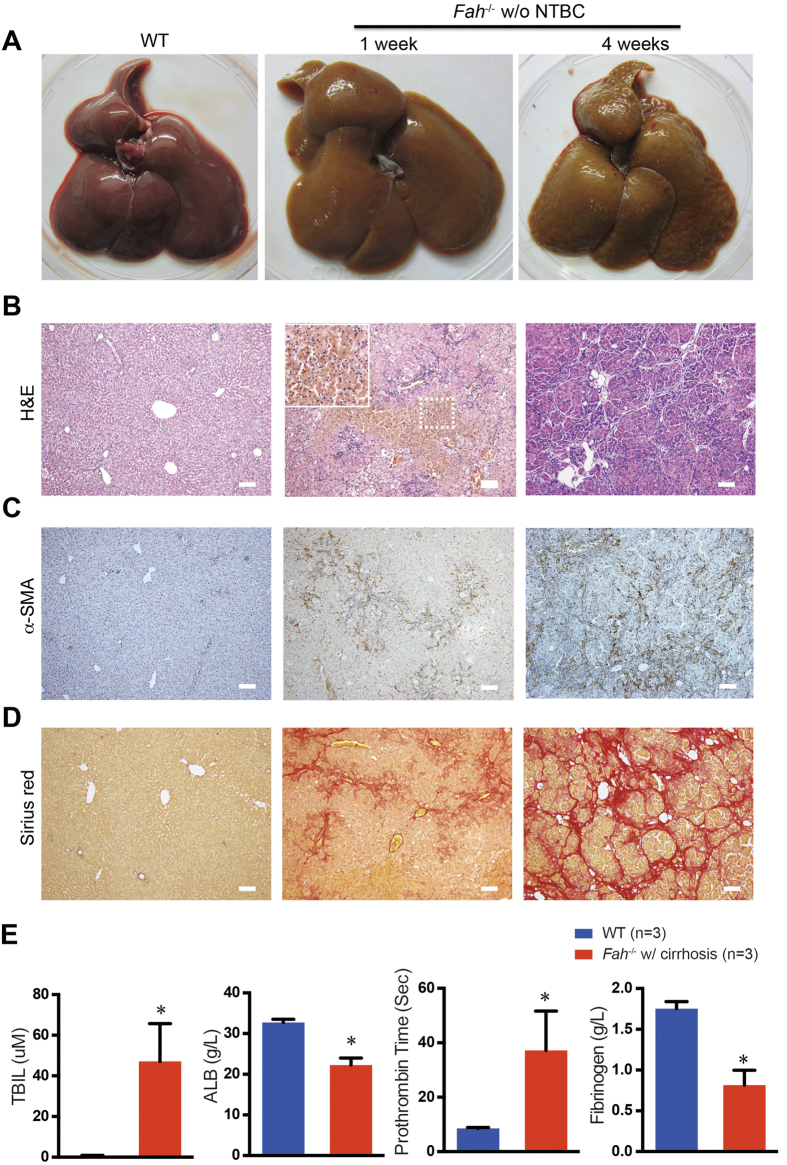
Liver necrosis and cirrhosis in *Fah*^−/−^ rats after NTBC withdrawal. (**A**) Livers of wild-type (WT) and *Fah*^−/−^ rats at 1 and 4 weeks after NTBC removal. The liver of a *Fah*^−/−^ rat at 4 weeks was firm with an irregular surface. (**B**) H&E staining of livers of WT and *Fah*^−/−^ rats at 1 and 4 weeks off NTBC. Impaired hepatocytes were enlarged in *Fah*^−/−^ rats. Liver necrosis and inflammation was observed. (**C,D**) Fibrosis and cirrhosis in *Fah*^−/−^ rat livers were determined by immunohistochemical staining for α-SMA (**C**) and Sirius red staining (**D**). WT rats receiving the same treatment were used as controls. Note that micronodules were surrounded by fibrous septa. Scale bar, 100 μm. (**E**) Plasma prothrombin time and fibrinogen levels and serum ALB and TBIL levels in WT (n = 3) and *Fah*^−/−^ (n = 3) rats with liver cirrhosis (*Fah*^−/−^ w/ cirrhosis). *P < 0.05, *t*-test.

In human HT1, liver injury is usually associated with diffuse necro-inflammation in the acute form and micro- and macro-nodular cirrhosis in the chronic form^6^. To further assess liver fibrosis and cirrhosis, we analyzed liver samples from *Fah*^−/−^ rats after NTBC withdrawal (Fig. 4A). Histopathological analyses of *Fah*^−/−^ livers at 1 week after NTBC withdrawal demonstrated massive necrosis associated with hepatocyte death and inflammatory cell infiltration (Fig. 4B). The extensive cell death was confirmed using TUNEL assay (Supplementary Fig. 5A). Elevated expression levels of several inflammatory cytokines were coincident with inflammatory cell infiltration (Supplementary Fig. 5B). Steatosis and bile duct hyperplasia were also observed in *Fah*^−/−^ livers (Supplementary Fig. 5C,D). Immunohistochemical staining of alpha smooth muscle actin (α-SMA), a marker of activated hepatic stellate cells, showed a large number of α-SMA-positive cells in *Fah*^−/−^ livers (Fig. 4C). Moreover, Sirius red staining revealed an increase in collagen deposition in the *Fah*^−/−^ livers (Fig. 4D). These findings suggest that the *Fah*^−/−^ rats rapidly developed liver fibrosis after NTBC withdrawal.

A small percentage of *Fah*^−/−^ rats survived more than 4 weeks after NTBC withdrawal (Fig. 3B). We then assessed whether fibrosis in *Fah*^−/−^ livers progressed to cirrhosis after prolonged liver injury. Livers from *Fah*^−/−^ rats at 4 weeks after NTBC withdrawal showed irregular external surface with nodules showing variable sizes and shapes, which is a typical feature of cirrhotic livers (Fig. 4A). Histopathological analysis showed a disruption of the structural organization of hepatic lobules, accompanied by the formation of pseudo-lobules (Fig. 4B). Furthermore, Sirius red staining demonstrated the formation of nodules by broad fibrous septa, which were not observed in WT rat livers (Fig. 4D), thereby suggesting prominent cirrhosis as assessed by the Scheuer system^38^. In line with liver cirrhosis, all surviving *Fah*^−/−^ rats developed ascites, and *Fah*^−/−^ rats showed symptoms of hepatic encephalopathy before dying, including fatigue, unresponsiveness, and drowsiness. Moreover, total bilirubin levels remained high in these rats, whereas their serum albumin levels decreased (Fig. 4E). The *Fah*^−/−^ rats also developed coagulopathy after NTBC withdrawal as revealed by prolonged prothrombin time (PT) and reduced fibrinogen (Fig. 4E). Taken together, the findings indicate that *Fah*^−/−^ rats develop severe liver injury and fibrosis after NTBC withdrawal, and those that survived longer than 4 weeks progressed to liver cirrhosis.

The kidney is another vital organ affected by HT1. Renal damage is generally variable and is mainly reflected in HT1 patients in the form of tubular dilatation, nephrocalcinosis, interstitial fibrosis, and involution of epithelial cells 6. The impaired kidney functions in *Fah*^−/−^ rats were indicated by elevated serum creatinine and blood urea nitrogen levels (Supplementary Fig. 6A). Histological analyses showed glomerulus inflammation, proximal tubule dilation, and epithelial cell detachment from tubules (Supplementary Fig. 6B). Moreover, Sirius red staining confirmed the existence of interstitial fibrosis (Supplementary Fig. 6C). These results indicate that *Fah*^−/−^ rats also develop kidney damage after NTBC withdrawal.

### Hepatocyte transplantation improves liver function and survival of *Fah* ^−/−^ rats

**Figure 5:**
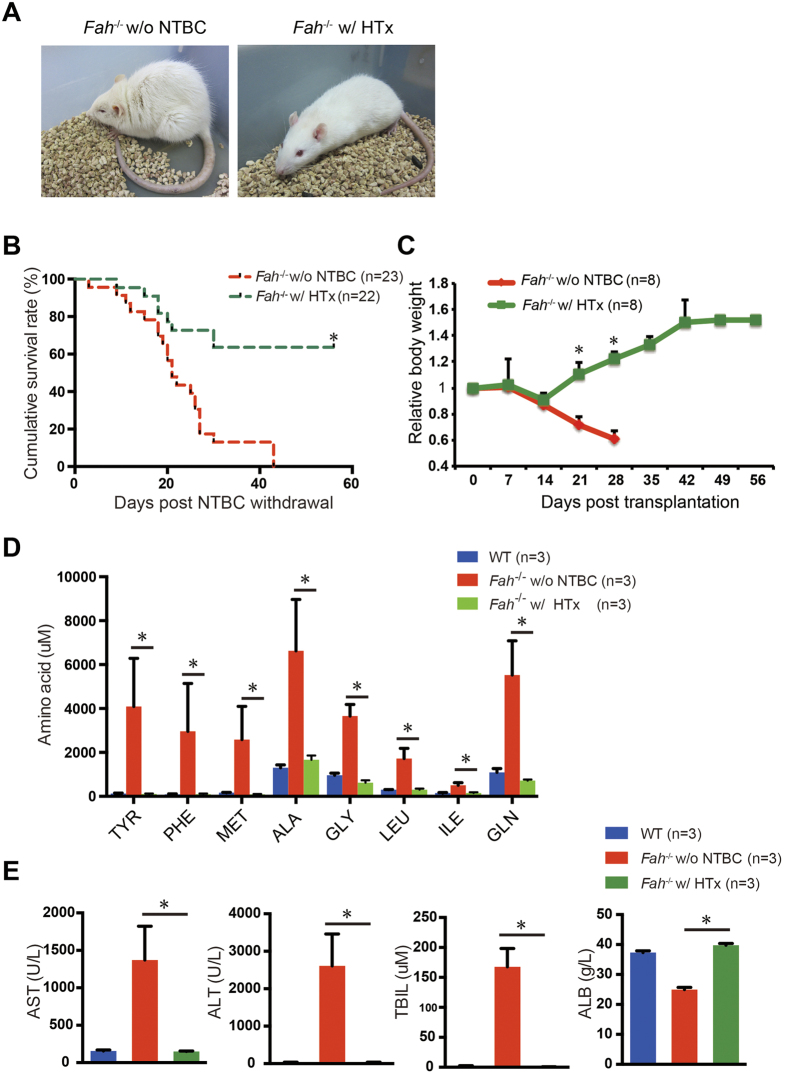
Improved liver function and survival of *Fah*^−/−^ rats after hepatocyte transplantation. Six- to eight-week-old *Fah*^−/−^ rats underwent WT hepatocyte transplantation (HTx). NTBC was withdrawn from drinking water immediately after HTx. (**A**) *Fah*^−/−^ rats after HTx at 4 weeks after NTBC withdrawal. *Fah*^−/−^ rats off NTBC for 3 weeks were used as control. Note that the control rat off NTBC was moribund, whereas the rat that underwent transplantation was healthy. (**B)** Kaplan-Meier survival curve of *Fah*^−/−^ rats with (n = 22) or without (n = 23) transplantation after NTBC removal. *P < 0.001, log-rank test. (**C**) The body weight of rats in both groups was measured every week. n = 8 for each group. *P < 0.01, *t*-test. (**D,E**) The serum levels of amino acids (**D**), ALT, AST, TBIL, and ALB (**E**) in WT (n = 3) and *Fah*^−/−^ rats with (n = 3) or without (n = 3) transplantation after NTBC removal. *P < 0.01, *t*-test.

*Fah*^−/−^ mice have been shown as one of best recipient animal models for hepatocyte transplantation^39^. We next characterized whether *Fah*^−/−^ rats could serve as a model for liver transplantation. NTBC administration was discontinued in 6–8-week-old *Fah*^−/−^ rats after receiving intrasplenic infusion of 1 × 10^7^ WT hepatocytes till they were sacrificed. The *Fah*^−/−^ rats that did not receive transplantation of WT hepatocytes showed a 40% decrease in body weight as expected and died within 7 weeks of NTBC withdrawal (Fig. 5A,B). In contrast, WT hepatocyte transplantation significantly improved the survival of *Fah*^−/−^ rats (Fig. 5A,B, 14 out of 22 rats survived). The rats transplanted with hepatocytes presented a 15% reduction in body weight during the first two weeks, but regained this later (Fig. 5C). The altered serum levels of tyrosine and other amino acids returned to their normal levels upon transplantation of WT hepatocytes (Fig. 5D). The serum ALT, AST, and TBIL levels of these rats also returned to their normal levels, and serum ALB levels increased, reaching levels comparable to that of WT rats (Fig. 5E).

**Figure 6:**
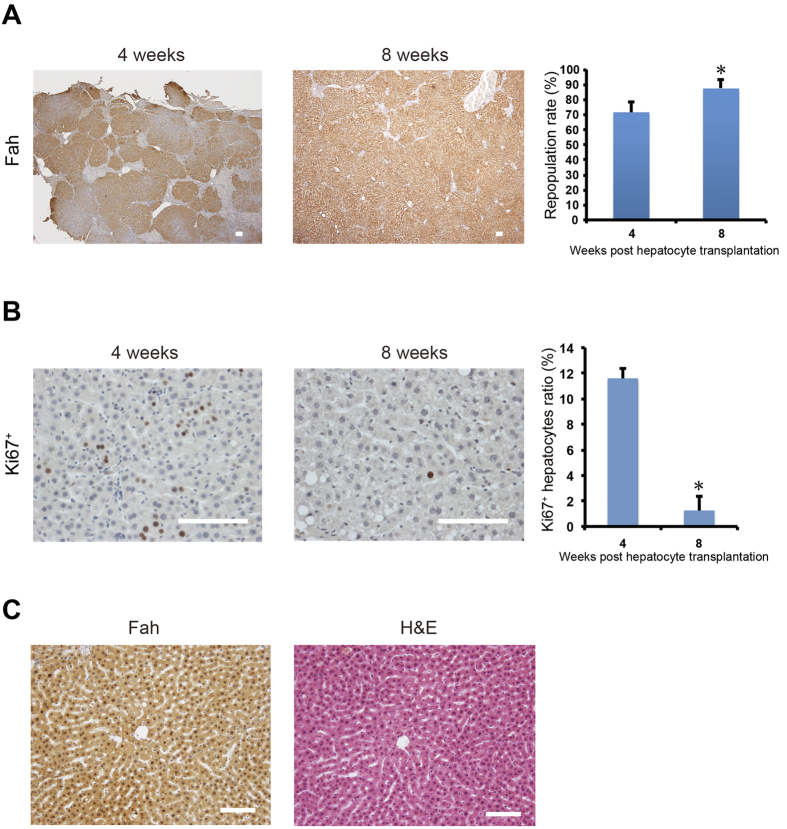
Repopulation of donor hepatocytes in *Fah*^−/−^ rat livers after transplantation. (**A**) Approximately 1 × 10^7^ WT hepatocytes from littermates were transplanted into *Fah*^−/−^ rats. Liver repopulation was analyzed at 4 (n = 3) and 8 (n = 3) weeks by immunohistochemical (IHC) staining for Fah. The repopulation rate was quantified. *P < 0.05, *t*-test. (**B**) Hepatocyte proliferation at 4 (n = 3) and 8 (n = 3) weeks after transplantation was determined by the IHC staining of Ki67. The ratio of Ki67-positive hepatocytes was quantified. *P < 0.01, *t*-test. (**C**) IHC staining of Fah and hematoxylin and eosin staining of serial liver sections from *Fah*^−/−^ rats at 8 weeks after transplantation. Scale bar, 100 μm.

To analyze repopulation efficiency, we harvested liver samples from *Fah*^−/−^ rats at 4 and 8 weeks after transplantation. Immunohistochemical staining with a Fah antibody showed extensive liver repopulation with 70% tissue replacement in recipients at 4 weeks after transplantation (Fig. 6A). Ki67 staining showed a high proliferation index at this time point (Fig. 6B). More than 90% liver repopulation was achieved at 8 weeks (Fig. 6A). Remarkably, Ki67 staining indicated a cessation of proliferation of transplanted cells in livers with near-complete repopulation of transplanted hepatocytes (Fig. 6B). The extensive cell death was induced in *Fah*^−/−^ livers after NTBC withdrawal (Supplementary Fig. 5A). 4 weeks after transplantation, cell death was reduced and only existed in recipient liver tissues but not in nodules repopulated by transplanted WT hepatocytes (Supplementary Fig. 5A). Therefore, the death of *Fah*^−/−^ hepatocytes might give repopulation advantage to the transplanted cells. HE staining confirmed that Fah-positive hepatocytes were morphologically normal (Fig. 6C). Together, these findings suggest that WT hepatocytes efficiently repopulated the entire liver and restored liver function in *Fah*^−/−^ rats, thereby indicating that *Fah*^−/−^ rats may be utilized as a biofactory for the expansion of transplanted hepatocytes.

### Progression to liver cirrhosis is prevented by hepatocyte repopulation

**Figure 7:**
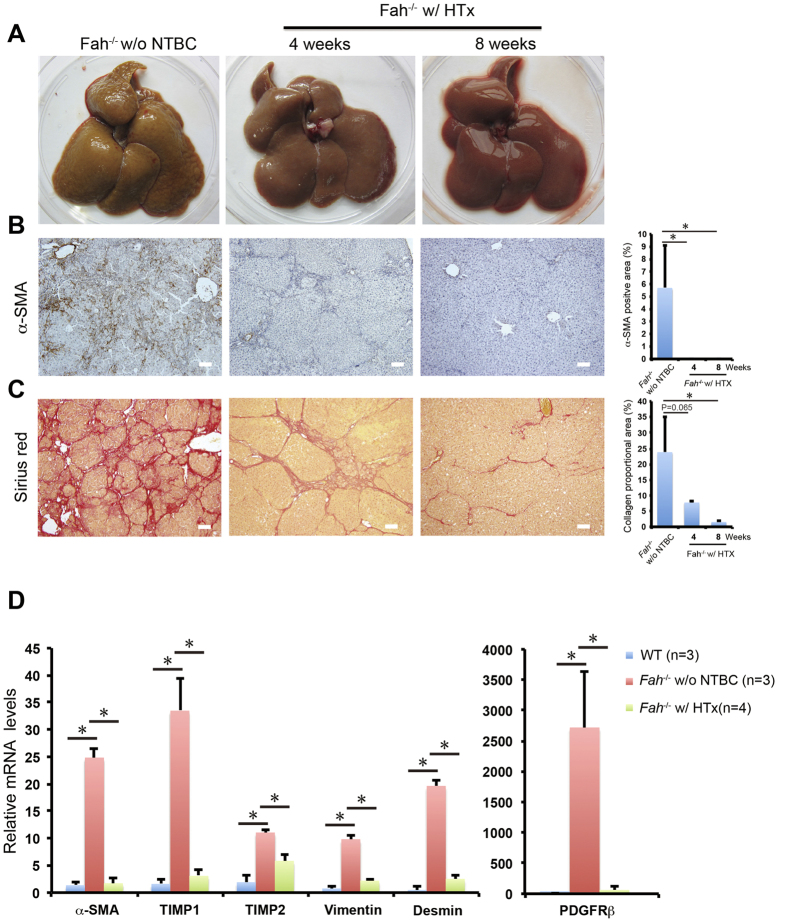
Attenuated liver cirrhosis in *Fah*^−/−^ rats after transplantation. *Fah*^−/−^ rats that underwent hepatocyte transplantation (*Fah*^−/−^ w/ HTx) were sacrificed at 4 and 8 weeks. *Fah*^−/−^ rats off NTBC for 3 weeks were used as control. (**A**) The livers of *Fah*^−/−^ w/ HTx or *Fah*^−/−^ w/o NTBC rats. (**B,C**) Liver fibrosis in *Fah*^−/−^ rats with (n = 3) or without (n = 3) HTx was evaluated by the immunohistochemical staining of α-SMA (**B**) and Sirius red staining (**C**). Quantification of α-SMA-positive area and Sirius red-stained collagen are shown in the right. *P < 0.05, *t*-test. (**D**) The mRNA levels of genes relative to stellate cell activation and fibrogenesis were determined by qPCR analysis of livers of WT (n = 3) and *Fah*^−/−^ rats with (n = 4) or without (n = 3) hepatocyte transplantation. *P < 0.05, *t*-test. Scale bar, 100 μm.

Because cirrhosis developed in *Fah*^−/−^ rats after NTBC withdrawal (Fig. 4), we determined whether repopulation of WT hepatocytes could ameliorate progression to liver cirrhosis. Compared to *Fah*^−/−^ livers without hepatocyte transplantation, WT hepatocytes that repopulated *Fah*^−/−^ livers were apparently soft and did not show irregular nodules macroscopically at 4 weeks. These macroscopic changes in *Fah*^−/−^ livers were more conspicuous at 8 weeks (Fig. 7A). mRNA levels of α-SMA, PDGFRβ, TIMP1, TIMP2, vimentin, and desmin were significantly upregulated in the livers of *Fah*^−/−^ rats at 4 weeks after NTBC withdrawal, which was apparently due to fibrogenesis (Fig. 7D). However, the expression of fibrogenic genes was dramatically reduced after WT hepatocyte transplantation (Fig. 7D). Furthermore, immunohistochemical staining of α-SMA indicated a reduction in the number of activated hepatic stellate cells in recipient livers at 4 and 8 weeks (Fig. 7B). Sirius red staining showed that collagen deposition in Fah^−/−^ rat livers was not significantly reduced [Collagen proportional area (CPA) = 7.8 ± 0.4%] at 4 weeks after hepatocyte transplantation, compared to those without transplantation (CPA = 24 ± 11%). However, collagen deposition was remarkably resolved at 8 weeks (CPA = 1.5 ± 0.3%; Fig. 7C). Histological analysis revealed larger nodules in WT hepatocyte transplanted *Fah*^−/−^ rat livers at 4 weeks (Fig. 7C). In contrast, *Fah*^−/−^ rat livers that did not undergo hepatocyte transplantation mainly presented small fragmented nodular cirrhosis (Fig. 7C). The resolution of collagen resulted in near-normal liver architecture in hepatocyte-transplanted *Fah*^−/−^ rat livers at 8 weeks (Fig. 7C). Taken together, WT hepatocyte repopulation reduced fibrogenic activity, and progression to liver cirrhosis was largely prevented after hepatocyte transplantation in *Fah*^−/−^ rats.

Finally, we analyzed whether repopulation of WT hepatocytes had any effect on kidney damage in *Fah*^−/−^ rats. Serum creatinine and blood urea nitrogen levels significantly decreased (Supplementary Fig. 7A). HE staining revealed a reduction in the number of dilated proximal tubules (Supplementary Fig. 7B), indicating that kidney function improved after correction of impaired liver function. However, interstitial fibrosis was still detectable by Sirius red staining (Supplementary Fig. 7C), suggesting renal tubular damage appears to be irreversible after hepatocyte transplantation.

## Discussion

*Fah*^−/−^ mice and pigs are invaluable models for mimicking major manifestations of HT1. Nevertheless, both models do not develop cirrhosis, the chronic presentation of HT1. Rats have recently been a research target of interest because of advances in genetic editing technology. We used the CRISPR/Cas9 system to generate Fah knockout rats. The *Fah*^−/−^ rats showed a reduction in body weight, failed to thrive, died due to progressive liver failure combined with other complications after NTBC withdrawal, and recapitulated the common presentation of human HT1. The significantly elevated serum levels of ALT, AST, TBIL, and other amino acids in *Fah*^−/−^ rats were indicative of severe liver damage. Massive hepatocyte death was also confirmed by histological analyses. Remarkably, typical liver cirrhosis was developed as shown by macroscopic changes in the liver, histological analyses, and laboratory biochemical examination. Furthermore, renal tubular damage was detected in *Fah*^−/−^ rats after NTBC withdrawal.

Despite the observed similarities, *Fah*^−/−^ mice, pigs and rats also have several differences. First, *Fah*^−/−^ rat embryos developed to term even without NTBC administration, which is similar to Fah-deficient human and mice, whereas Fah knockout pigs are embryonic lethal^12^. Second, compared to *Fah*^−/−^ mice and pigs, NTBC withdrawal induced higher serum levels of ALT, AST, and TBIL in *Fah*^−/−^ rats, thereby suggesting more severe liver injury in rats. It has been reported that transplantation of primary mouse hepatocytes to *Fah*^−/−^ mice leads to a survival rate of 100%^17^,^40^. However, around 60% *Fah*^−/−^ rats transplanted with WT hepatocytes survived. Interestingly, in transplantation to *Fah*^−/−^ mice, the survival and repopulation of human hepatocytes and iMPC-heps are increased by cycle NTBC off and on^18^,^22^. Therefore, it would be worth testing whether cycle NTBC off and on would improve the survival of transplanted *Fah*^−/−^ rats. Third, cirrhosis and bile duct hyperplasia were evident in *Fah*^−/−^ rats, which were not observed in *Fah*^−/−^ mice and pigs. Fourth, *Fah*^−/−^ mice developed liver cancer at the age of 10 months even on a standard dose of NTBC treatment^16^. In contrast, liver cancers were not detected in *Fah*^−/−^ rats at the age of 1 year under NTBC treatment (Supplementary Table 2).

Transplanted WT hepatocytes efficiently repopulated the livers of *Fah*^−/−^ mice and restored its functions^41^. We confirmed this finding in *Fah*^−/−^ rats. Though spontaneous correction of Fah mutations was reported in HT1 patients, it has been demonstrated that somatic reversion was not found in *Fah*^−/−^ mice^17^. A spontaneous reversion in *Fah*^−/−^ rats is unlikely, because this requires an in-frame insertion of 68 bp nucleotides in exon 2. Moreover, the present study showed that hepatocyte transplantation in *Fah*^−/−^ rats during the onset of liver injury effectively prevented the progression to liver cirrhosis. Previous studies have shown that hepatocyte transplantation reduces the extent of fibrosis in moderate liver fibrosis models such as lasiocarpine-treated rats, *Mdr2*^−/−^ mice, and the LEC rat model of Wilson’s disease^42^,^43^,^44^. However, it is more challenging to transplant hepatocytes into cirrhotic livers^45^. In rats with decompensated cirrhosis, hepatocyte transplantation prolonged survival time but transplanted cells appeared to lose function over time^46^. Another study also showed that transplanted hepatocytes repopulated livers with limited efficiency and did not improve the outcomes in rats with ccl4-induced cirrhosis^47^. In addition, a recent study characterized the transplantation of fetal liver stem cells (FLSCs) and mature hepatocytes in TAA-induced rat cirrhotic livers^48^. Transplantation of both types of cells resulted in more than 20% of liver repopulation, but did not significantly reverse the existing cirrhosis. Interestingly, transplantation of mesenchymal stem cells appeared to alleviate chemically induced liver fibrosis in rodents^49^,^50^,^51^. Nevertheless, whether cirrhosis could be fully reverted after extensive repopulation of transplanted hepatocytes remains elusive. Because *Fah*^−/−^ rats develop cirrhosis after NTBC withdrawal and permit the extensive repopulation of transplanted cells, it would be worth assessing the therapeutic efficacy of different types of cells, including hepatocytes, on liver cirrhosis in *Fah*^−/−^ rats.

Extensive liver humanization has been achieved by the transplantation of human hepatocytes into immune-deficient *Fah*^−/−^ mice^51^,^52^. Due to their small size, *Fah*^−/−^ mice only provides a relative low number of human hepatocytes. Immune-deficient *Fah*^−/−^ pigs are a desirable large animal that may be utilized as bioreactors for the large-scale production of human hepatocytes^13^; however, severe immune-deficient pigs are difficult to maintain^53^. Recently, immune-deficient rats have been generated under specific pathogen-free conditions^26^. Moreover, *Fah*^−/−^*IL2rg*^−/−^ rats have been used to evaluate the *in vivo* functions of rat liver stem cells^30^. *Fah*^−/−^Rag2^−/−^*IL2rg*^−/−^ (FRG) rats could be achieved by crossing *Fah*^−/−^*IL2rg*^−/−^ rats with *Rag*2^−/−^ rats. FRG rats may be potentially utilized as an alternative choice for the expansion of human hepatocytes. They could also be used as a model for the transplantation of liver progenitor cells and hepatocyte-like cells derived through direct differentiation or transdifferentiation^54^,^55^,^56^. Together, our study provides a novel rat model with the major features of HT1, including cirrhosis. *Fah*^−/−^ rats may be useful not only for the study of HT1, but also for hepatocyte transplantation.

## Methods

### Animal

All rats were maintained in specific pathogen-free husbandry. *Fah*^−/−^ rats were fed with drinking water containing 5.4 mg/L NTBC (Synthesized by Capot Chemical, China). All animal experimental protocols were approved by the Animal Care and Use Committee of Shanghai Institute of Biochemistry and Cell Biology. All animal research was carried out in accordance with the approved guidelines.

### Microinjection of sgRNA and Cas9 mRNA

Rat zygotes were obtained by superovulation of female SD rats (SLAC Shanghai) mating with the males. The zygotes were cultured in KSOM embryo culture medium (Millipore) before injection. TE solution containing 12.5 ng/uL of sgRNA and ~25 ng/uL of Cas9 mRNA was injected into the cytoplasm of one-cell stage embryos through the injection needle. Injected zygotes were transferred into pseudopregnant female rats immediately after injection.

### Founder identification by T7E1 mismatch sensitive assays

Tail clips were subjected to a standard DNA extraction procedure. The extracted DNA was amplified using the primers listed in Supplementary Table 3 to produce amplifications at a size of 733 bp around the target sites. After amplification was finished, the following program was carried out to generate mismatches into heteroduplexed DNA with PCR products generated from wild-type DNA: 98 °C, 5 min, 60 cycles of 98 °C, 30 sec, with auto-delta of 1 °C per cycle. The products were purified and 500 ng DNA per sample was digested with 0.3 ul of T7 endonuclease (New England Biotech) in a 10 ul reaction volume for 30 min at 45 °C. The mixture was then resolved on a 2% agrose gel.

### TA cloning and sequencing

To identify the modifications in founders, PCR products from the founder were cloned using TA cloning kit (Takara) following the manufacturer’s instructions. Four colonies were picked from each transformation and then sequenced with RV-M primer.

### Off-target site analysis

The off-target sites for each sgRNA were analyzed using a published prediction tool. A list of these target sites is provided in Supplementary Table 1. PCR primers were designed to flank the off-target sites as shown in Supplementary Table 3. The PCR products were directly sequenced.

### Hepatocyte isolation and transplantation

6–8 weeks rat were subjected to standard two-step collagenase perfusion for isolation of primary hepatocytes. Viability of isolated hepatocytes was around 90% as determined by Trypan blue. 1 × 10^7^ hepatocytes from WT littermates were transplanted through the spleen into *Fah*^−/−^ rats. NTBC was discontinued immediately after transplantation and all through the experiment. They were sacrificed at 4 and 8 weeks after transplantation. Liver and blood samples were collected for further analyses.

### Serum biochemical analysis

The rat blood was collected from the retro-orbital sinus and centrifuged at 12,000 r.p.m. for 5 min. The serum was frozen at −80 °C until biochemical analyses. Total bilirubin, albumin, ALT, AST, blood urea nitrogen and creatinine were measured by 7600-020 clinical analyser (Hitachi). Amino acids were quantified by liquid chromatographymass spectrometry ABI 3200 Q TRAP LC–MS/MS system (Applied Biosystem).

### Histology and immunohistochemistry

Liver tissues were collected immediately at the time rats were sacrificed. Liver samples were fixed by paraformaldehyde and embedded in paraffin. Sections were subjected to hematoxylin and eosin (H&E), immunohistochemistry (IHC), Sirius red staining according to the standard protocols. Antibodies used for IHC staining are as follows: Rabbit anti-Fah (AbboMax, San Jose, CA, 1:3000), Rabbit anti-Ki67 (novocastra, 1:1000), Rabbit anti-α-SMA (Abcam, 1:200), Rabbit anti-Ck19 (Abcam, 1:200).

### RNA extraction and quantitative PCR

Total RNA was isolated from liver samples by Trizol (Invitrogen). Quantitative PCR (qPCR) was performed with SYBR Premix Ex Taq (TaKaRa) on an ABI StepOne Plus real-time PCR system (Applied Biosystems). A list of the primers is shown in Supplementary Table 3.

### Statistics

For most statistic evaluation, an unpaired Student’s t test was applied for calculating statistical probability in this study. *P* values were calculated by two-tailed test. For survival analysis, the Mantel-Cox log-rank test was applied. Statistic calculation was performed using GraphPad Prism5 (GraphPad).

## Additional Information

**How to cite this article**: Zhang, L. *et al*. Efficient liver repopulation of transplanted hepatocyte prevents cirrhosis in a rat model of hereditary tyrosinemia type I. *Sci. Rep.* **6**, 31460; doi: 10.1038/srep31460 (2016).

## Supplementary Material

Supplementary Information
