# Supplementary material for: Efficient liver repopulation of transplanted hepatocyte prevents cirrhosis in a rat model of hereditary tyrosinemia type I
Source: Sci Rep. 2016 Aug 11;6:31460. doi: 10.1038/srep31460 (PMC4980609; doi:10.1038/srep31460)
Supplement: Supplementary Information [file srep31460-s1.pdf]

Efficient liver repopulation of transplanted hepatocyte prevents cirrhosis in a rat model of hereditary tyrosinaemia type I

Ludi Zhang, Yanjiao Shao, Lu Li, Feng Tian, Jin Cen, Xiaotao Chen,

Dan Hu, Yan Zhou, Weifen Xie, Yunwen Zheng, Yuan Ji, Mingyao Liu,

Dali Li and Lijian Hui

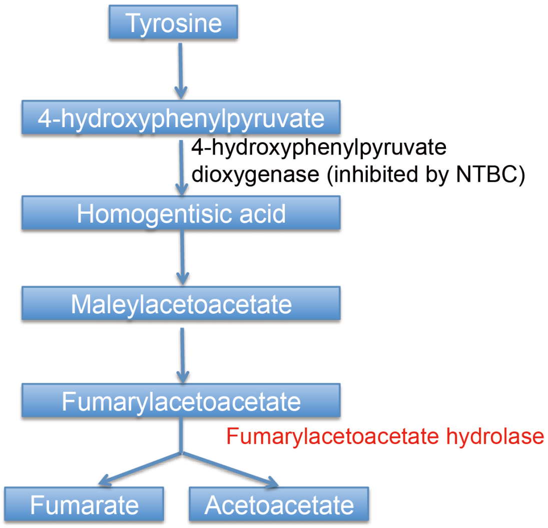

Supplementary Fig. 1. Schematic diagram of the tyrosine metabolism pathway

FAH is the last enzyme in the tyrosine metabolic pathway. FAH deficiency causes the accumulation of toxic metabolites, including fumarylacetoacetate and maleylacetoacetate. NTBC blocks upstream pathways and rescues liver damage.

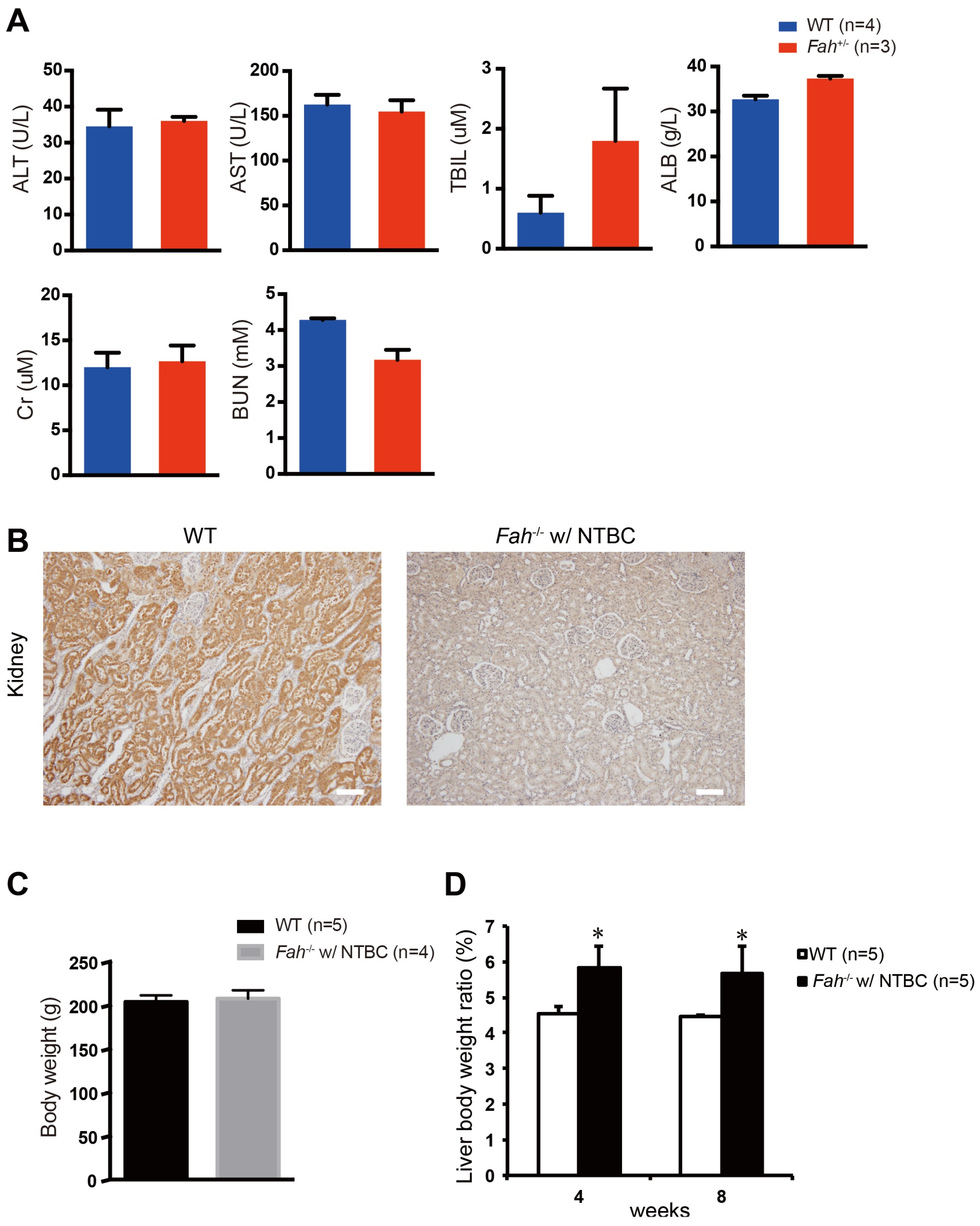

Supplementary Fig. 2. The phenotype of *Fah*$^{+/-}$ and NTBC treated *Fah*$^{-/-}$ rats

(**A**) The serum levels of ALT, AST, TBIL, ALB, Cr, and BUN in 2-month-old WT (n=4) and *Fah*$^{+/-}$ (n=3) rats. (**B**) Immunohistochemical staining of Fah in the kidneys of WT and *Fah*$^{-/-}$ rats. (**C**) The body weight of two-month-old *Fah*$^{-/-}$ (n=5) and WT (n=4) rats. (**D**) The liver body weight ratio of 4 (n=5)- and 8 (n=5)-week-old WT and *Fah*$^{-/-}$ rats. *, *P*<0.01, *t*-test.

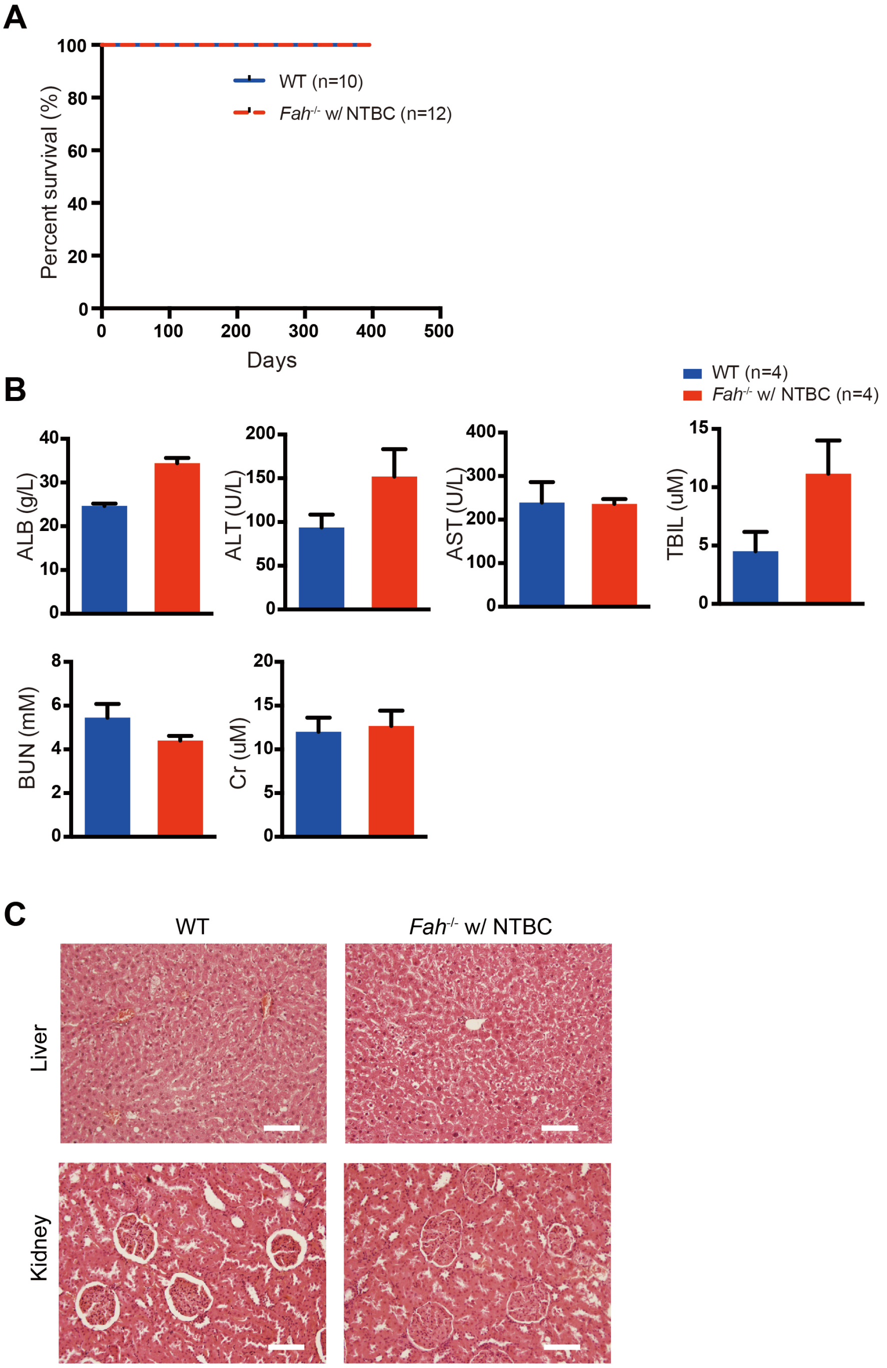

Supplementary Fig. 3. The long-term effect of NTBC treatment on *Fah*$^{-/-}$ rats

(**A**) Kaplan-Meier survival curve of WT (n=10) and *Fah*$^{-/-}$ (n=12) rats treated with NTBC for one year. (**B**) Serum ALT, AST, TBIL, ALB, Cr, and BUN levels of

one-year-old WT (n=4) and *Fah*$^{-/-}$ (n=4) rats. (**C**) Hematoxylin and eosin staining of the livers and kidneys of *Fah*$^{-/-}$ rats treated with 1 mg/kg/Day NTBC for one year.

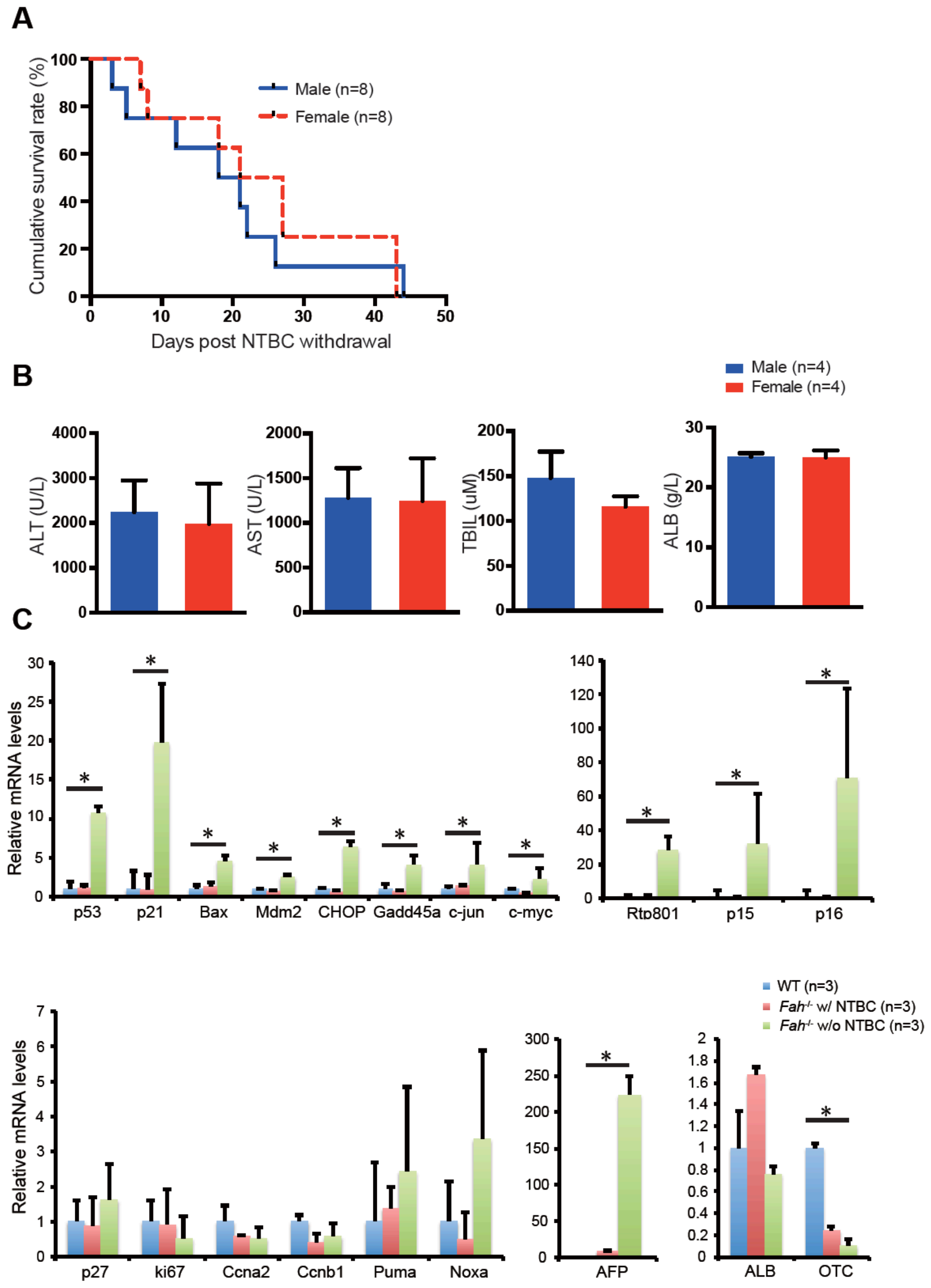

Supplementary Fig. 4. No significant sex differences among NTBC-off *Fah*$^{-/-}$ rats and effects of NTBC in liver mRNA expression

(**A**) Kaplan-Meier survival curve of male (n=8) and female (n=8) *Fah*$^{-/-}$ rats after NTBC withdrawal. (**B**) Serum ALT, AST, TBIL, and ALB levels of in male (n=4) and female (n=4) *Fah*$^{-/-}$ rats after NTBC withdrawal. (**C**) The expression levels of a subset of well-characterized genes in WT (n=3) and *Fah*$^{-/-}$ rats with (n=3) and without (n=3) NTBC.

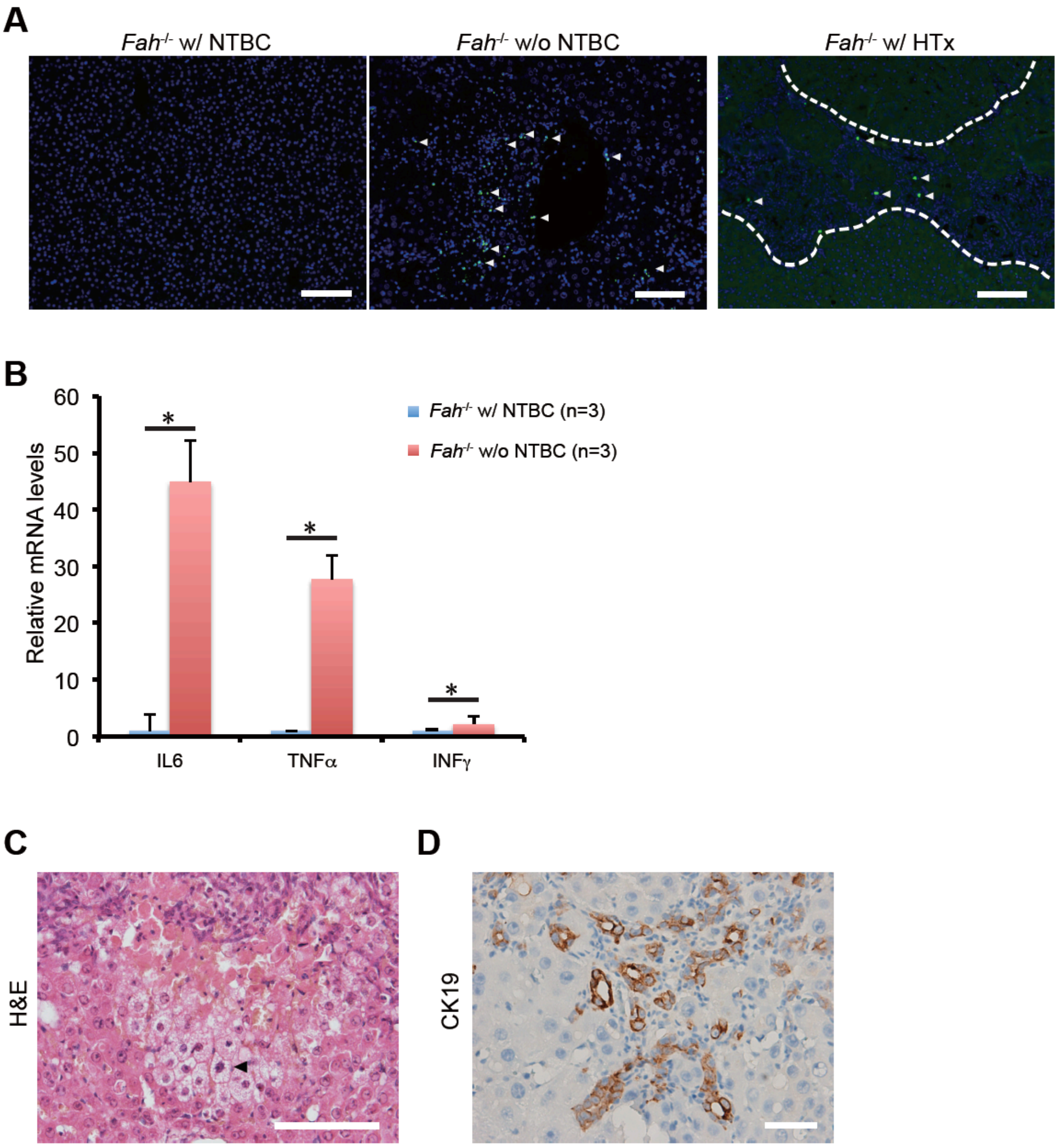

Supplementary Fig. 5. The pathology of the livers of *Fah*$^{-/-}$ rats after NTBC withdrawal

(**A**) The cell death was confirmed using TUNEL assay in WT rats, *Fah*$^{-/-}$ rats at one week after NTBC withdrawal and *Fah*$^{-/-}$ rats at one week after hepatocyte transplantation. (**B**) The gene expression levels of several inflammatory cytokines in WT (n=3) and *Fah*$^{-/-}$ (n=3) rats at one week after NTBC withdrawal. (**C**) The steatotic hepatocytes in *Fah*$^{-/-}$ rats off NTBC were identified by hematoxylin and eosin staining.

Black arrowheads indicate steatotic hepatocytes. (**D**) Bile duct hyperplasia in *Fah*$^{-/-}$ rats off NTBC was determined by immunohistochemical staining of Ck19.

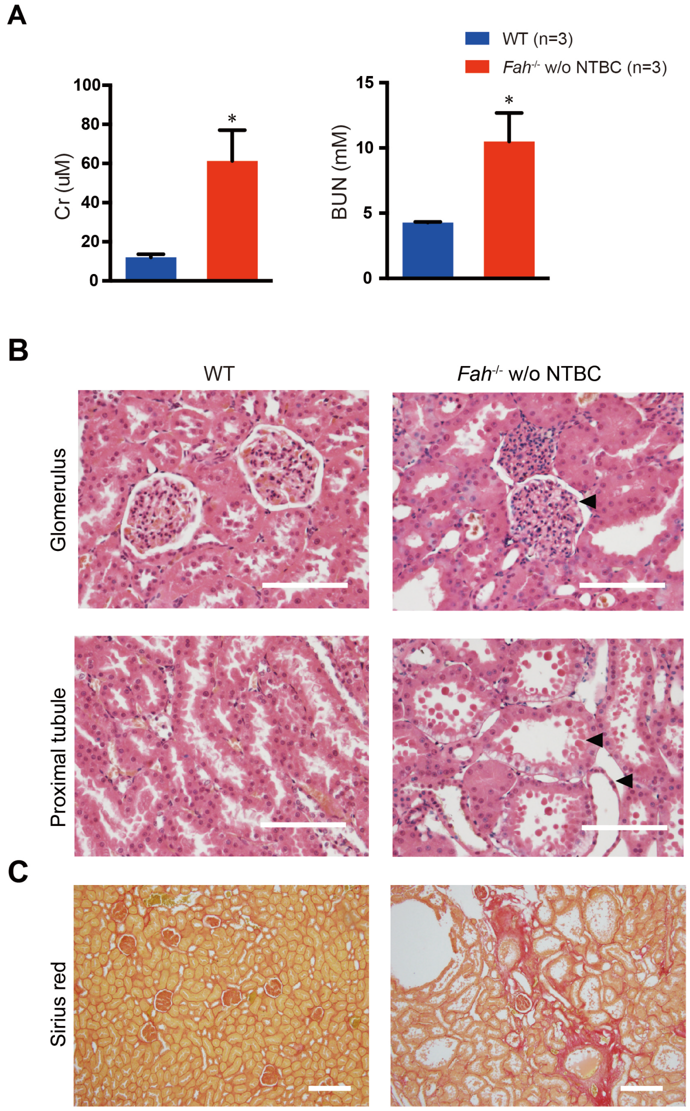

Supplementary Fig. 6. Renal damage in *Fah*$^{-/-}$ rats after NTBC withdrawal

(**A**) Serum Cr and BUN levels of WT (n=3) and *Fah*$^{-/-}$ (n=3)rats after NTBC withdrawal. *, P<0.01, t-test. (**B**) Hematoxylin and eosin staining of kidneys of WT and *Fah*$^{-/-}$ rats off NTBC. Black arrows indicate inflammation of the glomerulus

(upper panel), dilation of the proximal tubules, and involution of epithelial cells (lower panel) of kidneys of *Fah*$^{-/-}$ rats. (**C**) Sirius red staining of kidneys of WT and *Fah*$^{-/-}$ rats off NTBC. Interstitial fibrosis was observed in *Fah*$^{-/-}$ rats. Scale bar, 100 μm.

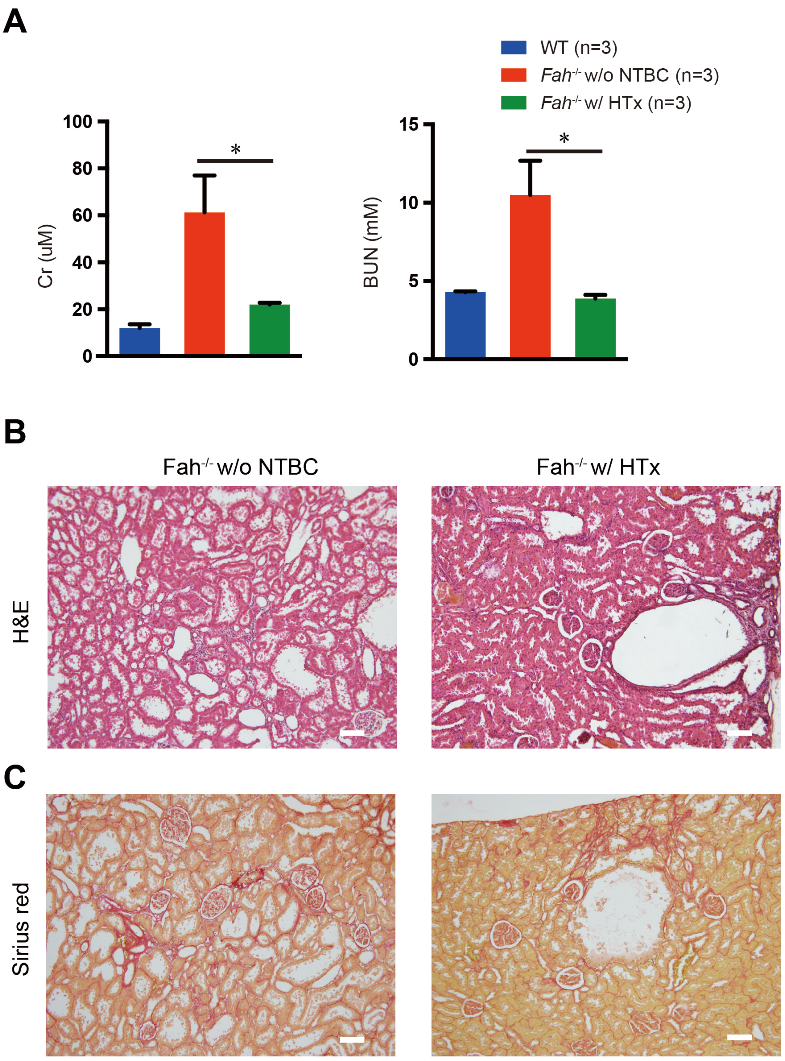

Supplementary Fig. 7. Improved kidney function in *Fah*$^{-/-}$ rats after hepatocyte transplantation

(**A**) Serum Cr and BUN levels of WT (n=3) and *Fah*$^{-/-}$ rats with (*Fah*$^{-/-}$ w/ HTx) (n=3)

or without (*Fah*$^{-/-}$ w/o NTBC) (n=3) transplantation. (**B-C**) Hematoxylin and eosin staining **(B)** and Sirius red staining **(C)** of kidneys of *Fah*$^{-/-}$ rats with or without transplantation.

ID	sequence	score	mismatches	UCSC gene	locus	Note
sgRNA1	ATCGAAGACATGCTGATGTTTGG	66	0MMs	Fah	chr1:-147659410	
1	AACAAAGGCATGCTGATGTTAGG	2.5	3MMs [2:4:8]		chr9:+37180501	Fah sgRNA1 off-target1
2	AATGAAGACCTGCTGATGTTGAG	2.4	3MMs [2:3:10]		chrX:+32555829	Fah sgRNA1 off-target2
3	CTCAAAGACATGCTGATGTACAG	1.5	3MMs [1:4:20]		chr7:+126789106	Fah sgRNA1 off-target3
4	CTGGAAGACAAGCTGATGTTAGG	1.5	3MMs [1:3:11]		chr16:+40575463	
5	ATCAGAGACATGGTGATGTTGGG	1.1	3MMs [4:5:13]		chr3:-154672944	
6	ATGGCAGACATGCTGATGATGGG	1	3MMs [3:5:19]		chr5:-66123640	
7	CTAGAACCCATGCTGATGTTAGG	0.9	4MMs [1:3:7:8]		chr10:+32390830	
8	TTGGAATCCATGCTGATGTTGGG	0.9	4MMs [1:3:7:8]		chr9:+49381297	
9	CTTGAACACCTGCTGATGTTTGG	0.9	4MMs [1:3:7:10]		chr15:-51614193	
10	CTCTGAGAGATGCTGATGTTTAG	0.9	4MMs [1:4:5:9]		chr20:+34573447	

ID	sequence	score	mismatches	UCSC gene	locus	Note
sgRNA2	CCACGGATTGGTGTGGCCATCGG	61	0MMs	Fah	chr1:+147659331	
1	CCAGGCATTGGTGTGGCCATGGG	3.3	2MMs [4:6]		chr10:+103218149	Fah sgRNA2 off-target1
2	CCAGGCATTGGTGTGGCCATGGG	3.3	2MMs [4:6]		chr10:-103286029	
3	CCAGGCATTGGTGTGGCCATGGG	3.3	2MMs [4:6]		chr10:+103518303	Fah sgRNA2 off-target2
4	CCAGGCATTGGTGTGGCCATGGG	3.3	2MMs [4:6]		chr10:+103367164	Fah sgRNA2 off-target3
5	CCAGGCATTGGTGTGGCCATGGG	3.3	2MMs [4:6]		chr10:-103307218	
6	CCAGGCATTGGTGTGGCCATGGG	3.3	2MMs [4:6]		chr10:-103235486	
7	CCAGGCATTGGTGTGGCCATGGG	3.3	2MMs [4:6]		chr10:+103436774	
8	CCAGGCATTGGTGTGGCCATGGG	3.3	2MMs [4:6]		chr10:-103149871	
9	CCAGGCATTGGTGTGGCCATGGG	3.3	2MMs [4:6]		chr10:+103385927	
10	CAAGGGATAGGTGTGGCCATTGG	1.6	3MMs [2:4:9]		chr10:+59218115	

Supplementary Table 1. The predicted off-target sites

Top 10 potential off-target sites for each *Fah* sgRNA in the rat genome. The score is the likelihood of off-target binding. The PCR products from loci highlighted in yellow were cloned and sequenced. No mutations in these potential off-target sites in the founder #15 were observed.

	Fah$^{-/-}$ rat	*Fah*$^{-/-}$ mouse	*Fah*$^{-/-}$ pig
Embryonic lethal	No	No	Yes
Serum biochemical parameters	ALT >1000 U/L AST >900 U/L TBIL>100 uM	ALT and AST<500 U/L TBIL<80uM	ALT and AST<500 U/L TBIL<80uM
liver cirrhosis	Yes	No	Not reported
bile duct proliferation	Yes	No	Not reported
liver tumor	Not found at age 12 months	Found at age 10 months	Not reported

Supplementary Table 2. Comparison among *Fah*$^{-/-}$ rats, mice, and pigs

Gene	Primer sequence	Length (bp)
**Primers for genotyping**		
Fah	F 5’-CTGCTGCATTTAAGCTACCAC-3’<br>R 5’-CATAGAAGCCAGGATGAGTGT-3’	733
**Primers for off-target anlysis**		
Fah sgRNA1 Off-target1	F 5’-CACTGAAGTCTGGGAAGGCAAAT-3’<br>R 5’-AGCCTGAGGTTGGGAAATGC-3’	778
Fah sgRNA1 Off-target2	F 5’-AAAATCCCACTGAATTGCTAG-3’<br>R 5’-GGAAATCCAAACCCTTCTACC-3’	721
Fah sgRNA1 Off-target3	F 5’-GGAAGGCAAATCCCACTGAC-3’<br>R 5’-ACAAGAGGGAGATCCAAACC-3’	735
Fah sgRNA2 Off-target1	F 5’-AAACATCACTGCAAAGGTAA-3’<br>R 5’-GTACAATGAAAGAAAGGGTG-3’	680
Fah sgRNA2 Off-target2	F 5’-CCATTCTGCTAGGAGGGTGC-3’<br>R 5’-CCTGTCTGGTGCTGGGATTA-3’	601
Fah sgRNA2 Off-target3	F 5’-CTCAAGGAACATCACAGGAA-3’<br>R 5’-TTGGAAGAAAAGCAATCAAA-3’	716
**Primers for qPCR**		
TIMP1	F 5’-AGCCTGTAGCTGTGCCCCAA-3’<br>R 5’-AACTCCTCGCTGCGGTTCTG-3’	252
TIMP2	F 5’-GGATTCCGGGAATGACATCTAT-3’<br>R 5’-CGCCTTCCCTGCAATTAGATA-3’	147
α-SMA	F 5’-TGTGCTGGACTCTGGAGATG-3’<br>R 5’-GAAGGAATAGCCACGCTCAG-3’	148
Vimentin	F 5’-AATGCTTCTCTGGCACGTCT-3’<br>R 5’-GCTCCTGGATCTCTTCATCG-3’	100
PDGFRβ	F 5’-GAGACTGACAATGACTACATC-3’<br>R 5’-GGCTGTCGCAGGAGATGGT-3’	103
Desmin	F 5’-GTGAAGATGGCCTTGGATGT-3’<br>R 5’-CGGGTCTCAATGGTCTTGAT-3’	109

p53	F 5’-GCGTTGCTCTGATGGTGA-3’<br>R 5’-CAGCGTGATGATGGTAAGGA-3’	232
p21	F 5’-GCAAAGTATGCCGTCGTCT-3’<br>R 5’-CAAAGTTCCACCGTTCTCG-3’	111
MDM2	F 5’-GGTCTATCGGGTCACAGTCT-3’<br>R 5’-TCTCACGAAGGGTCCAACAT-3’	161
Bax	F 5’-TAGCAAACTGGTGCTCAAGGC-3’<br>R 5’-GGGTCCCGAAGTAGGAAAGG-3’	152
AFP	F 5’-ATGAGTAGCGATGCGTTGGC-3’<br>R 5’-GGAAAGTGGAAGGGTGGGAC-3’	218
Alb	F 5’-CAAGAGCCCGAAAGAAACGA-3’<br>R 5’-CTGGCAACTTCATGCAAATAGTGT-3’	152
OTC	F 5’-CAATATCCTGCACTCCATCA-3’<br>R 5’-TCTTCTCATCCTCTTGTCCC-3’	230
CHOP	F 5’-ACCTTCACTACTCTTGACCCTG-3’<br>R 5’-CTCATTCTCCTGCTCCTTCTC-3’	231
p15	F 5’-CATCCCTAAACACGAACCTATC-3’<br>R 5’-ACCAATGAAACAGTGCCAAA-3’	214
p16	F 5’-GTCGTGCGGTATTTGCGGTAT-3’<br>R 5’-CTCGCGTTGCCAGAAGTGAA-3’	181
p27	F 5’-TGGAGAAGCACTGCCGAGAT-3’<br>R 5’-CAGAGTTTGCCTGAGACCCAAT-3’	243
Ki67	F 5’-TTTAAGAAGTTCAGGGCTACA-3’<br>R 5’-CTCTTTCCTACTTTGGGTGA-3’	290
CCNA2	F 5’-TTTGCCATCGCTTATTGCTG-3’<br>R 5’-GTGGTGCTTTGAGGTAGGTCTG-3’	158
CCNB1	F 5’-ATAAAGTCAGCGAACAGTCAA-3’<br>R 5’-CAAGAATCACATCGGAGAAA-3’	279
Gadd45a	F 5’-CAGCGAGGCTAAGCAAGAAG-3’<br>R 5’-TACACGCCGACAGTTATGGT-3’	249
Apaf1	F 5’-ACCAGAGGACGTGGAGGTGA-3’<br>R 5’-ACTGCCAAATGGTCGTAGGG-3’	189

Rtp801	F 5’-ACCTTTCAGTTGACCCTGGTGC-3’<br>R 5’-GCTCGGAGCTGTAGAGTTTCTTTT-3’	163
Puma	F 5’-GCACTGATGGAGATACGGACTTGG-3’<br>R 5’-AGCCTTTCCTGAGATGGTGGTG-3’	138
Noxa	F 5’-GCAGAGTTACCGCCTGAATT-3’<br>R 5’-TCTTCTCATCGTGCTCTTTCG-3’	138
Jun	F 5’-GTCTACGCCAACCTCAGCAACTT-3’<br>R 5’-CGTCTGCGGCTCTTCCTTCA-3’	198
MYC	F 5’-GAGGAGAAACGAGCTGAAGCG-3’<br>R 5’-TGAACGGACAGGATGTAGGC-3’	126
TNFa	F 5’-GGAAAGCATGATCCGAGATG-3’<br>R 5’-CAGTAGACAGAAGAGCGTGGTG-3’	145
IL6	F 5’-TTGCCTTCTTGGGACTGATG-3’<br>R 5’-ACTGGTCTGTTGTGGGTGGT-3’	100
INFγ	F 5’-TGAACAACCCACAGATCCAG-3’<br>R 5’-AATCAGCACCGACTCCTTTT-3’	109

Supplementary Table 3. List of primer sequences used in the study
